# Evaluation and Characterization of Acute respiratory distress syndrome in tree shrews through TMT proteomic method

**DOI:** 10.1371/journal.pone.0319752

**Published:** 2025-04-16

**Authors:** Junlong Xiong, Liji Zhang, Jinchao Xing, Weijian Huang, Ning Wang, Xiaoyan Lin, Shuhua He, Ming Liao, Jun He

**Affiliations:** 1 State Key Laboratory for Animal Disease Control and Prevention, South China Agricultural University, Guangzhou, China; 2 Key Laboratory of Livestock Disease Prevention of Guangdong Province, Key Laboratory for prevention and control of Avian Influenza and Other Major Poultry Diseases, Ministry of Agriculture and Rural Affairs, Institute of Animal Health, Guangdong Academy of Agricultural Sciences, Guangzhou, China; 3 South China Normal University, Guangzhou, China; 4 Institute of Laboratory Animal Science, Jinan University, Guangzhou, China; 5 Zhongkai University of Agriculture and Engineering, China; University of Michigan, UNITED STATES OF AMERICA

## Abstract

Acute respiratory distress syndrome (ARDS), a common cause of acute fatal respiratory, is characterized by severe inflammatory lung injury as well as hallmarks of increased pulmonary vascular permeability, neutrophil infiltration, and macrophage accumulation. Tree shrew, a squirrel-like small animal model, has been confirmed to have more similar traits to human ARDS with one-hit intratracheal instillation of LPS in our previous study. In this study, we characterized protein profile changes induced by intranasal LPS challenge in the tree shrew model through tandem mass tag (TMT)-based quantitative proteomics and type II alveolar epithelial cells through pathological analysis. In total, 4070 proteins (*p* <  0.05) were identified from lung tissues of the LPS-induced group and PBS group. Among the differential expression proteins (DEPs) detected by *t*-*t*est (≥|1.5-fold|), 529 DEPs were identified, of which 304 were upregulated, and 225 were downregulated. The most important pathways involved in the process of ARDS had been identified by enrichment analysis: oxidative stress, apoptosis, inflammatory responses, and vascular endothelial injury. In addition, proteins have been reported in animal models or clinical patients also detail investigated for further analysis, such as ceruloplasmin (CP), hemopexin (HPX), sphingosine kinase 1 (SphK1), lactotransferrin (LTF), and myeloperoxidase (MPO) were upregulated in induced tissues and confirmed by western blot analysis. Overall, this study not only reveals a comprehensive proteomic analysis of the ARDS tree shrew model but also provides novel insights into multi-pathways responses induced by the LPS challenge of tree shrews. We highlight shared and unique proteomic changes in the lungs of ARDS tree shrews and identify novel pathways for acute lung injury, which may promote the model into basic research and translational research.

## 1. Introduction

Acute respiratory distress syndrome (ARDS) is a dangerous fatal respiratory associated with an acute-onset, widespread, inflammatory form of lung injury that leads to hypoxemic respiratory insufficiency and even death [1]. The characteristics of human ARDS are uncontrolled and sustained alveolar inflammation, pulmonary vascular endothelial injury, oxidative injury, and progression of apoptosis, which represented pulmonary edema, neutrophil infiltration and macrophages accumulation [2–4]. To elucidate the mechanisms underlying progression during the complex pathophysiology of ARDS, animal models are important to solve and develop therapeutic approaches of diseases [5,6].

As a novel animal model, the Chinese tree shrew (*Tupaia belangeri chinensis*)—a low-primate small mammal with many phenotypic and genomic features that are valuably similar to humans—has been used to establish various models of human disease, especially those multiple subtypes of viral hepatitis that only occur in humans [7–16]. More importantly, because of its similar immune responses of lungs to humans, it has been widely used as a disease model in respiratory system, which includes influenza virus infection, SARS-CoV-2 infection, and lung fibrosis [9,17–20]. Previously, in line with the New Berlin Definition of human ARDS, we have successfully established a tree shrew model sensitized to ‘one-hit’ lipopolysaccharide (LPS) intratracheal instillation, which exhibited unique advantages in obvious hypoxemia, formation of a hyaline membrane and intensive inflammatory infiltration in the lung [21].

In this study, we have investigated the molecular changes that occur in the lung tissues of tree shrews induced with LPS. We have conducted a proteomics analysis to identify related pathways and characterize the traits of pathological lesions and comprehensively evaluated the protein profiles with changed levels in important pathways such as immunity, apoptosis, oxidative stress, and pulmonary vascular endothelial injury. These findings provide an original insight into the changes that take place during the process of lung injury in the lung tissues of tree shrews. This translational research can pave the way for the development of treatments for ARDS and promote the use of this novel animal model in studying the pathogenesis of human ARDS.

## 2. Materials and methods

### 2.1 Ethics statement and animals

All animal experimental protocols were approved by the Institutional Animal Care and Use Committee of Jinan University (Approval No. IACUC-20191014-01). Relevant research work was carried out in accordance with the “Regulations on the Administration of Laboratory Animals in Guangdong Province” adopted by the Standing Committee of the Guangdong Provincial People’s Congress. Tree 2-year-old tree shrews were from the Animal Experimental Centre of Kunming Medical University, China. They were reared in individual self-designed cages (Application CN103461160A, China) and were housed under controlled temperature (25 ~ 28°C), humidity (50 ~ 70%), lighting (12:12 h light-dark cycle) conditions and provided with free access to sterile water and food.

### 2.2 LPS-induced lung injury model

The experimental design and protocol used were based on a previous study [21]. The reporting of data was compliant with the ARRIVE guidelines in the reporting of *in vivo* animal research [22]. According to the previous design, two independent experimental groups were conducted, totaling 12 animals, with a PBS group (n = 6 tree shrews per group) and an LPS group (n = 6 tree shrews per group). Animals are randomly assigned to each group. PBS group–Tree shrews were exposed to PBS and euthanized with sodium pentobarbital [90 mg/kg, intraperitoneal (i.p.)] after 120 hours. When clinical symptoms were observed in the LPS-induced group, such as breathing difficulties, tree shrews were euthanized with sodium pentobarbital [90 mg/kg, intraperitoneal (i.p.)]. Each group had two lung tissues mixed together as one sample, and thus, there were three samples in each group. All samples were collected for histopathological examination, TMT analysis and western blot analysis. All tree shrews were obtained from the Animal Experimental Centre of Kunming Medical University, China. Each experimental operation has a fixed operator to ensure the accuracy of the experiment.

### 2.3 Histopathological analysis

The fixed lung tissues were dehydrated and continually embedded in paraffin and cut into 4 µm-thick slices. Immunofluorescence (IF) staining of surfactant protein C (SP-C) and aquaporin 5 (AQP5) was performed on the number of changes in alveolar epithelial cells. The commercial antibodies used for IF were purchased from Servicebio Technology Company (GB113318, GB113682). Panoramic digital slide SCAN (3D Histech, Hungary) was used to scan the digital slide images, and Case Viewer (Version 2.4, 3D Histech Ltd., Hungary) was used to evaluate. Histopathological analysis was performed as previously described [23]. According to a previous study, Image Pro Plus software (Version 6.0, Media Cybernetics, Inc., USA) was used to calculate the number of changes in inflammatory cells and alveolar epithelial cells, as previously mentioned [21].

### 2.4 TMT analysis

#### 2.4.1 Protein preparation.

The six samples of each group were dispensed into 3 Eppendorf (EP) tubes. According to the standardized protocol, tissue was homogenized by grinding in liquid nitrogen and diluted in 500 µ L of SDT buffer (pH 8.0, 100 mM Tris-HCl, 4% SDS, and 100 mM DTT) into each EP tube [24]. The tube was then heated at 100°C for 10 minutes, sonicated on ice for 5 minutes, and finally centrifuged at room temperature (RT) 14000 × g for 30 minutes. 1 µ L of each sample was quantified by the bicinchoninic acid method. The remaining lysate was frozen at -80°C until use.

#### 2.4.2 Trypsin digestion.

Protein digestion was executed using the filter-aided sample preparation (FASP) procedure [25]. Loaded 300 µg of proteins into a solution containing 200 μL of UA buffer (pH 8.0, 8 M urea, 150 mM Tris-HCl, China) on the ultrafiltration filter (30 kDa cutoff, Sartorius, Germany), centrifuged at 14,000 × g for 15 min and washed UA buffer three times (100 μL). To block the reduced cysteine residue, UA buffer solution (100 μL) containing 50 mM iodoacetamide was added to the filter. The samples were incubated at RT in the dark for 30 min and then centrifuged at 14,000 × g for 10 min. The filters were washed by UA buffer (200 μL) three times and centrifuged at 14,000 × g for 10 min for each time. Next, 100 μL of dissolution buffer (Applied Biosystems, Foster City, USA) was added to each filter, centrifuged at 14,000 × g for 10 min, and repeated three times. Then, trypsin buffer (52 μL, Promega, USA) was used to digest protein suspensions at 37°C for 18 h. At last, the filter unit was transferred to a new tube, and 40 μL of dissolution buffer was added. Then, it was centrifuged at 14,000 × g for 30 min. The obtained peptides were collected as filtrate, and the concentration of peptide was analyzed at OD280.

#### 2.4.3 Off-line high-pH reversed-phase fractionation.

Serial samples from each group and samples were included in one TMT6 labeling experiment. Merge TMT-labeled peptides were equipped with a Gemini-NX (Phenomenex, 00F-4453-E0, USA) column (4.6 × 150 mm, 3 µm, 110 Å), subjected to High-pH Reversed-Phase Fractionation in 1100 Series HPLC Value System (Agilent, USA), and eluted at a flow rate of 0.8 mL/min [26]. Buffer A was composed of 10 mM Ammonium acetate (pH 10.0), and Buffer B was composed of 10 mM Ammonium acetate (pH 10.0), 90% v/v acetonitrile (ACN). All those buffers have been filtered and sterilized. The gradient for separation consisted of several steps: (1) 100% Buffer A for 40 min; (2) Mixed 95% Buffer A and 5% Buffer B for 7 min; (3) Mixed 70% Buffer A and 30% Buffer B for 20 min; (4) Mixed 30% Buffer A and 70% Buffer B for 30 min; (5) Mixed 25% Buffer A and 75% Buffer B for 32 min. Monitored the elution process by measuring absorbance at 214 nm and collected fractions every 1.25 min. The approximately 40 collected fractions were ultimately merged into 12 pools. Concentrated each fraction through vacuum centrifugation and reconstituted in 10 μL of 0.1% v/v formic acid. All samples were stored at -80°C until subjected to LC-MS/MS analysis.

#### 2.4.4 LC-MS/MS.

The Easy-nLC nanoflow HPLC system was connected to Orbitrap Elite mass spectrometer (Thermo Fisher Scientific, USA) to analyze TMT-labeled samples. An autosampler was used at a flow rate of 200 nL/min, and 1μg of each sample was loaded onto the Thermo Scientific EASY column (two columns). Peptides in the Thermo Scientific EASY trap column (100 μm × 2 cm, 5 μm, 100 Å, C18) and analytical column (75 μm × 25 cm, 5 μm, 100 Å, C18) were captured. Before each analysis, rebalance the column to its initial high aqueous solvent composition. The mass spectrometer was operated in positive ion mode, and MS spectra were in the range of 350-2000 m/z. The resolution of the Orbitrap Elite’s MS scan and MS/MS scan at 100 m/z were set as 60,000 and 15,000, respectively. The most intense signals were selected from MS spectra for further MS/MS analysis. The isolation window was 2 m/z, and ions underwent high-energy collisional dissociation and fragmentation, with a normalized collision energy of 35 eV. The maximum ion implantation times for investigation scanning and MS/MS scanning were set to 10 ms and 100 ms, respectively, and the automatic gain control target values for the full scanning model were set to 1e^6^ and 5e^4^, respectively. The duration of dynamic exclusion was 30 s.

#### 2.4.5 Database search.

The Proteome Discoverer 2.3 software (Thermo Fisher Scientific with the Mascot search engine) was used to analyze all raw files. The MASCOT search engine (Matrix Science, London, UK; Version 2.3) embedded in Proteome Discoverer was used to search fragment spectrum in the Uniprot_Tupaia_chinensis_20893_20190903.fasta (20,893 sequences). The following search parameters were used: (1) trypsin as the cleaving enzyme; (2) monoisotopic mass; (3) TMT 6 plex labeling of lysine and peptide N-term and methionine oxidation as variable modifications; (4) two missed cleavages; (5) peptide charges of 2 + , 3 + , and 4 + ; (6) cysteine carbamidomethylation was used as fixed modifications.

The mass tolerance of precursor ions was set to 20 ppm, and the mass tolerance of fragment ions was set to 0.1 Da. A 1% error detection rate was set for Peptide spectral matching. The relative quantitative analysis of sample proteins was based on the ion ratios reported by TMT, which represented all unique peptides of each protein. The relative peak intensities of the TMT-reported ions were obtained in each of the MS/MS spectra, and a normalized final ratio was obtained from relative protein quantification according to the median average protein quantification ratio. The ProteomeXchange Consortium stored the mass spectrometry proteomics data through an iProX partner repository with the dataset identifier PXD053328 (http://proteomecentral.proteomexchange.org) [27].

#### 2.4.6 Bioinformatics and Enrichment Analysis.

The relative analysis was performed as previously mentioned [24]. Use the rstatix R package (version 1.42.2) to perform data correction on the proteome data, and screen differential proteins with *p*.adjust <  0.05 (*p* value FDR correction, algorithm selection BH) and | log2 (FC) |>  log2 (1.5) (DEPs). Download the Chinese tree shrew genome and proteome sequences from the website (http://www.treeshrewdb.org/). Then, use the eggNOG 6.0 website (http://eggnog6.embl.de/) [28] to annotate the proteome, and the resulting annotation files were used in the AnnotationForge R package (version 1.42.2) to create the OrgdbR package of the Chinese tree shrew. For differential genes, the constructed Chinese tree shrew OrgdbR package was used in the Cluster Profiler software package with q value <  0.05 as the statistical significance criterion to perform Gene Ontology (GO) [29] and Kyoto Encyclopedia of Genes and Genomes for differential genes. Encyclopedia (KEGG) [30] enrichment analysis. The Chinese tree shrew PPI network was constructed based on the STRING database (high confidence, https://string-db.org, version 12.0) [31]. Then, the MCODE plug-in in Cytoscape (version 3.10.1) was applied to identify modules in the PPI network based on the vertex-weighting scheme.

#### 2.5 Western blots.

Lung tissues were homogenized in 1 × RIPA buffer (containing cocktail protease inhibitors, ST506, Beyotime Biotechnology Co., Ltd., China) on ice for 20 min and then at 4°C centrifuged at 15,000 × g for 20 min. The supernatant was observed and stored at -80°C. Western blot analysis was performed, as previously mentioned [24]. SDS-PAGE was used to separate the equal amounts of proteins, which were transferred onto a polyvinyl fluoride membrane (1620177, Bio-Rad, USA). After blocking with 5% BSA, the primary antibody (anti-ceruloplasmin, 55156-1-Ig, Proteintech, USA; anti-hemopexin A5603, Abclonal, USA; anti-sphingosine kinase 1, 10670-1-AP, Proteintech, USA; anti-lactotransferrin, A12902, Abclonal, USA; anti-myeloperoxidase, A1374, Abclonal, USA; anti-plasminogen activator inhibitor 2, 16035-1-AP, Proteintech, USA; anti-β-Tubulin, KTD101-CN, Abbkine, USA) was incubated at 4°C overnight. Membranes were washed in TBST containing Tween20 and incubated at RT for 2 h with the secondary antibody (1:2,000; Cell Signaling Technologies, USA). The membranes were visualized with an Odyssey system. The band intensity was quantified using Image J software.

#### 2.6 Statistical analyses.

All data processing and analysis were performed in RStudio (version 4.3.2). To compare two groups of continuous variables, the statistical significance of normally distributed variables was calculated using an independent Student’s *t*-test, and differences between non-normally distributed variables were calculated using the Mann-Whitney U-test (i.e., Wilcoxon rank-sum test). The chi-square test or Fisher’s exact test was carried out to analyze the statistical significance between two sets of categorical variables. Correlation coefficients between different genes were estimated via Pearson correlation analysis. All statistical *p* values were two-sided, and *p* <  0.05 was considered statistically significant.

## 3. Results

### 3.1 One-hit LPS administration induced lung hyper-responsiveness

**Fig. 1:**
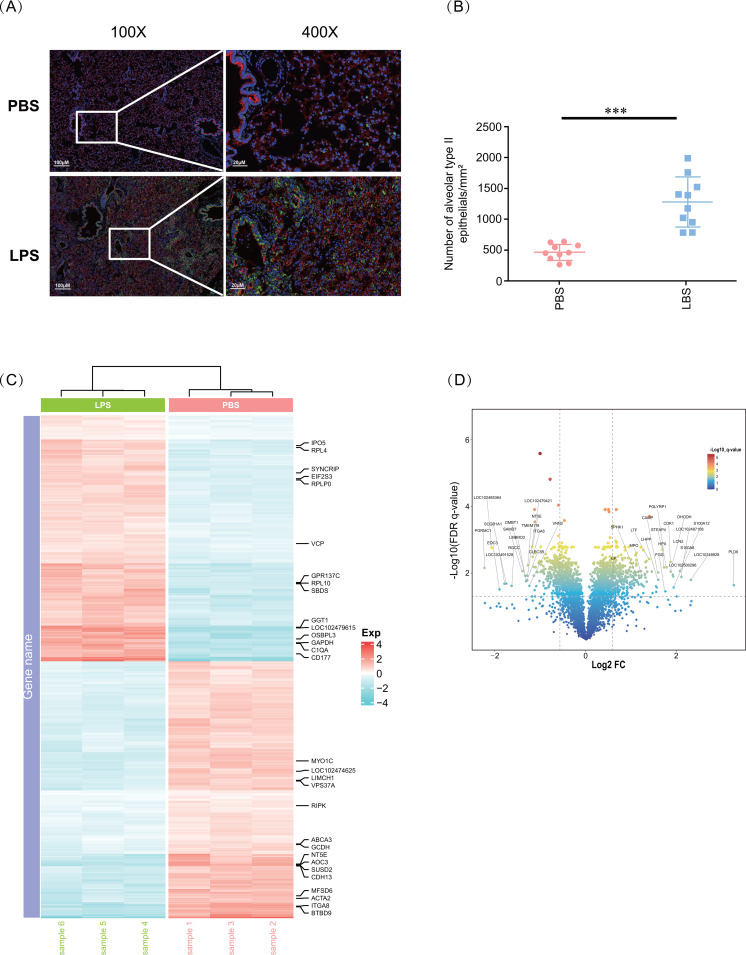
LPS induces lung hyper-responsiveness in tree shrews. (A) Immunofluorescence of images of the PBS and LPS groups. Representative images of the two groups at magnifications of 100 × and 400 × , stained immunofluorescence with aquaporin 5 (AQP5) antibody and surfactant protein **C** (SP-C) antibody and DAPI. Alveolar type I epithelial cells were identified with red fluorescence, while alveolar type II epithelial cells were identified with green fluorescence. The DAPI stained the nucleus blue. The LPS group showed obvious increased alveolar type II epithelial cells. (B) Semi-quantize analysis of the slides. The number of alveolar type II epithelial cells was calculated based on slides. (C) Differential expression heatmap between PBS and LPS, respectively. Three biological replicates are shown. (D) Heatmap of genes corresponding to differentially expressed proteins (DEPs). The heatmap displays 775 differentially expressed genes (DEGs) selected through statistical testing (*t*-test, *p*.adjust <  0.01), highlighting the most significant differential genes. To emphasize significance, only the top 30 genes with the smallest *p*.adjust values are shown in the figure, representing the most differentially expressed genes. The read indicated upregulated genes, and blue showed downregulated genes. ***p <  0.001 vs PBS.

In order to gain insight into the ARDS-related changes in lung tissues in ARDS tree shrews induced by LPS, we investigated the type II alveolar epithelial cells associated with alveolar permeability and protein profile in their lung tissues at the endpoint [33]. In our previous study, the main histopathological changes involved obvious inflammatory cell infiltration, especially neutrophils and macrophages, which are mainly located in the alveolar cavity and alveolar septum [21,32]. These data showed that the numbers of type II alveolar epithelial cells were dramatically increased in the LPS-induced tree shrew model, which is consistent with other animal models (Fig 1A and Fig 1B) [34–36].

A TMT-based quantitative proteomic method was used to evaluate lung injury and to analyze lung-specific proteomics, which is considered more decisive and capable than proteomics from other organs [37]. The purpose was to find reliable and valuable biomarkers for pathogenesis, diagnosis, and prognosis. In this study, LPS-induced lung proteomics was conducted to explore different signaling pathways, potent DEPs, and biomarkers. The study used statistical analysis (*t*-test with *p*.adjust <  0.05), and protein abundances were selected if they were | log2 (FC) | >  log2 (1.5) [38,39]. A total of 4070 proteins were identified and quantified using MaxQuant (S1 Table), of which 529 DEPs were identified, 304 were upregulated, and 225 were downregulated (S2 Table). The heatmap displays gene expression data corresponding to DEPs. Each data point represents the expression level of a gene and its corresponding protein expression difference. As shown in Fig 1C, A total of 775 differentially expressed genes are shown in the heatmap, which exhibits significant expression differences in the samples analyzed. Hierarchical cluster analysis revealed that the samples achieved a standard of technical replicate variance (Fig 1D).

### 3.2 Administration of LPS changed protein profiles in the lung of tree shrews

**Fig. 2:**
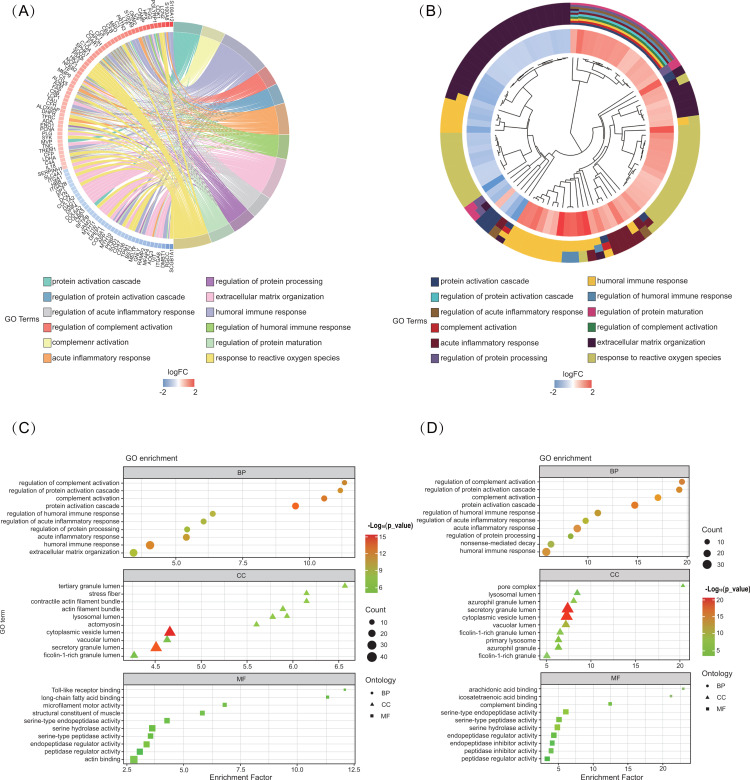
Functional enrichment analysis of DEPs. (A) A chord dendrogram of GO cluster plot. GO cluster plot showing a chord dendrogram of the clustering of the expression spectrum of significantly changed genes. (B) A circular dendrogram of GO cluster plot. GO cluster plot showing a circular dendrogram of the clustering of the expression spectrum of DEPs. (C) GO term enrichment dot plot, triangle diagram, and square of the (C) DEPs and **(D)** upregulated genes. The y-axis represents GO‑enriched terms. The x-axis represents the fold of enrichment.

**Fig. 3:**
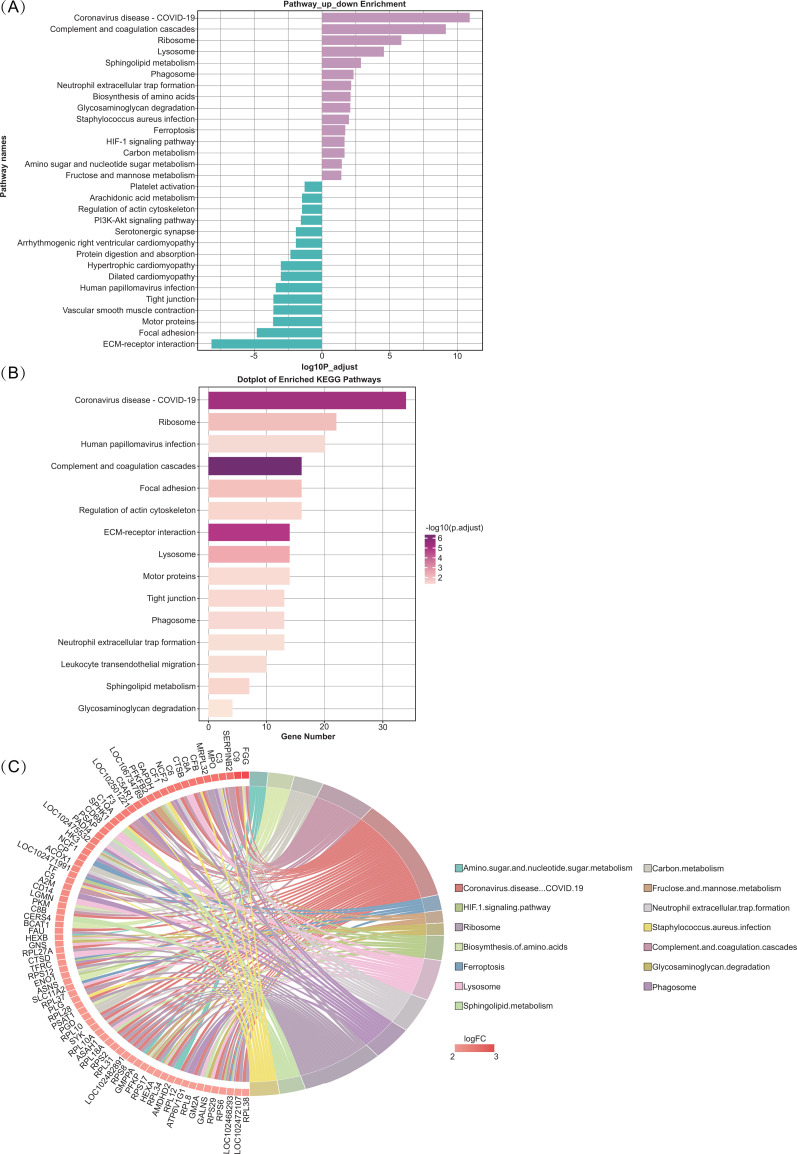
KEGG analysis of DEPs. (A) Top 30 enriched of KEGG enrichment. (B) KEGG pathway analysis of upregulated DEPs. (C) The circos diagram of the DEPs and its corresponding KEGG signaling pathways.

Gene ontology (GO) and KEGG enrichment analysis were performed to identify the functional characteristics of the DEPs. For MF, the DEPs were enriched in ‘actin binding’ (GO: 0003779, involving 25 genes), ‘peptidase regulator activity’ (GO: 0061134, involving 15 genes), ‘serine hydrolase activity’ (GO: 0017171, involving 15 genes), ‘actin filament binding’ (GO: 0051015, involving 12 genes), ‘endopeptidase inhibitor activity’ (GO: 0004866, involving 12 genes). Regarding CC, the DEPs were enriched in ‘cytoplasmic vesicle lumen’ (GO: 0060205, involving 41 genes), ‘secretory granule lumen’ (GO: 0034774, involving 38 genes), ‘external side of plasma membrane’ (GO: 0009897, involving 21 genes), ‘extrinsic component of membrane’ (GO: 0019898, involving 21 genes) and ‘endocytic vesicle’ (GO: 0030139, involving 21 genes). The GO analysis results revealed that the upregulated DEPs were significantly enriched in ‘humoral immune response’ (GO: 0006959, involving 35 genes), ‘extracellular matrix organization’ (GO: 0030198, involving 29 genes), ‘response to reactive oxygen species’ (GO: 0000302, involving 26 genes), ‘protein processing’ (GO: 0016485, involving 24 genes) and ‘acute inflammatory response’ (GO: 0002526, involving 22 genes). Notably, the upregulated DEPs were enriched in ‘humoral immune response’, ‘acute inflammatory response’ and ‘protein activation cascade’ in terms of BP. For CC, the upregulated DEGs, significant enrichment was observed in the ‘secretory granule lumen’, ‘cytoplasmic vesicle lumen’ and ‘vacuolar lumen’. Under MF, the DEPs were enriched in ‘serine-type endopeptidase activity’, ‘serine hydrolase activity’ and ‘endopeptidase regulator activity’ (Fig 2 and S3 Table). In addition, KEGG pathway enrichment analysis was performed to determine the biological processes and functions of the 1.5-fold DEPs. There were the top 30 (up- and down- the top 15 pathways) KEGG pathways in Fig 3: the Coronavirus disease-COVID-19 (34 genes), Complement and coagulation cascades (16 genes), Ribosome (22 genes), ECM-receptor interaction (14 genes) and Neutrophil extracellular trap formation (13 genes).

### 3.3 LPS challenge increases the immune accumulation and ARDS-related pathways

**Fig. 4:**
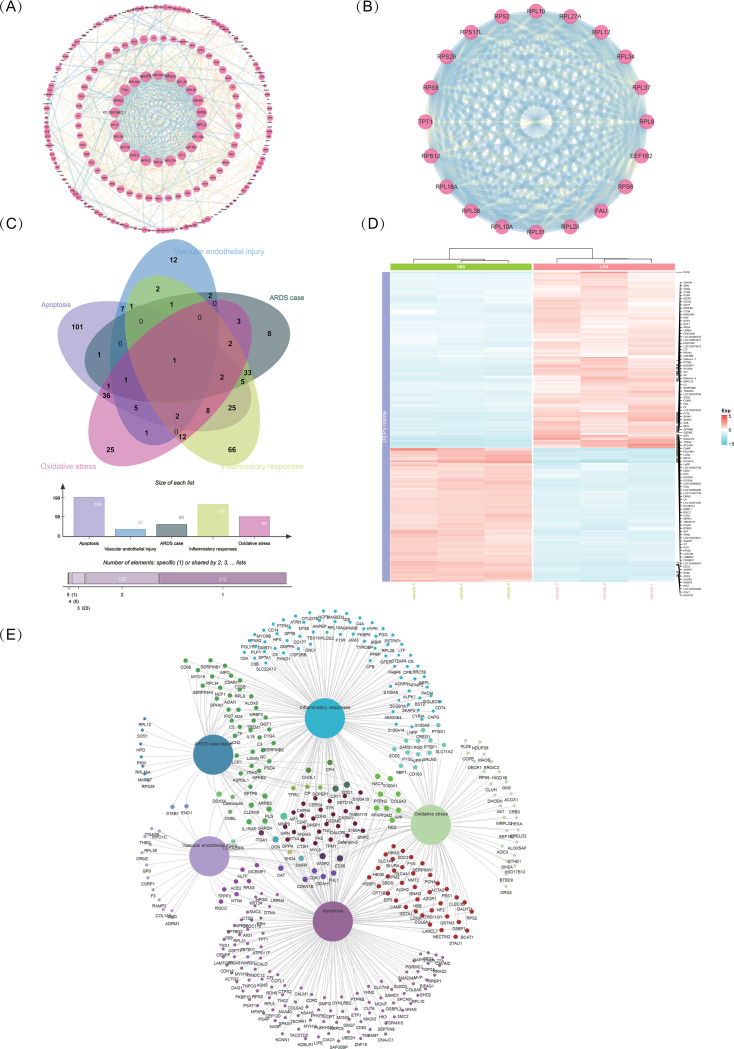
Constructed protein-protein interaction (PPI) networks and conducted analysis. (A) Construction of PPI networks based on STRING database data. The circle size represents the MCODE score, where a higher score indicates higher connectivity and dense interactions within the module. These modules typically represent functionally related groups of proteins. A lower score indicates looser connections within the module, potentially signifying weaker functional relationships among these nodes. (B) Selection of high-scoring modules from the STRING network analysis. (C) Identification of key areas involving oxidative stress, immunity, apoptosis, endothelial damage, and ARDS case reports, with 1.5-fold DEPs. A Venn diagram shows the overlapping human proteins across the five categorized groups. (D) A heatmap plotted using the pheatmap R package (version 1.42.2). It displays relative expression levels with intensities of red and blue, indicating the relative increase and decrease of these proteins. (E) Integrated PPI network analysis of the five categorized groups. PPI network analysis was performed using Cytoscape based on the STRING database with high confidence for each category group. Constructed oxidative stress PPI network, consisting of 99 nodes and 199 edges. Constructed apoptosis PPI network, consisting of 192 nodes and 314 edges. Constructed endothelial damage PPI network, consisting of 35 nodes and 71 edges. Constructed inflammatory response PPI network, consisting of 160 nodes and 282 edges. Constructed ARDS case report-related PPI network, consisting of 63 nodes and 129 edges.

Determination of the PPI network offered a conceptual framework through which to better explore the functional organization of the disease-related DEPs and their potential interactions (Fig 4A, S4 Table). PPI network analysis, conducted using the STRING database, showed a tight interaction network of all the identified proteins at the high confidence score of 0.9, as displayed in Fig 4B. Lung injury and inordinate inflammation were induced by different sorts of proteins interacting with each other and participating in a series of ARDS-induced and ARDS-caused related pathways and interactive proteins in lung tissue [40]. Therefore, we focused on the DEPs in the LPS-induced group, conducting functional annotation based on information from databases and literature in relation to their role in pathogenesis. A total of the 392 DEPs (among 1.5-fold DEPs) were classified into 5 functional groups according to the suggested functional annotation relationship, immunity mechanism and ARDS pathogenesis, including (1) Proteins related to oxidative stress response, (2) Proteins involved in apoptosis process, (3) Proteins related to vascular endothelial damage process, (4) Immune system related proteins and (5) Proteins reported in ARDS patients or animal models (Fig 4C, 4E). Heatmaps of differential expression results of the 392 DEPs are shown in Fig 4D (S3 Table). As shown in Fig 4, 112 DEPs were involved in the oxidative stress group, 170 DEPs were involved in the inflammatory response, 38 DEPs were involved in the vascular endothelial damage process, 202 DEPs were involved in the apoptosis group, and 68 DEPs were involved in the ARDS models. Notably, a significant number of DEPs (170) were related to immune response—including interleukin-1 (IL-18).

### 3.4 Validation of selected proteins from proteomic data

**Fig. 5:**
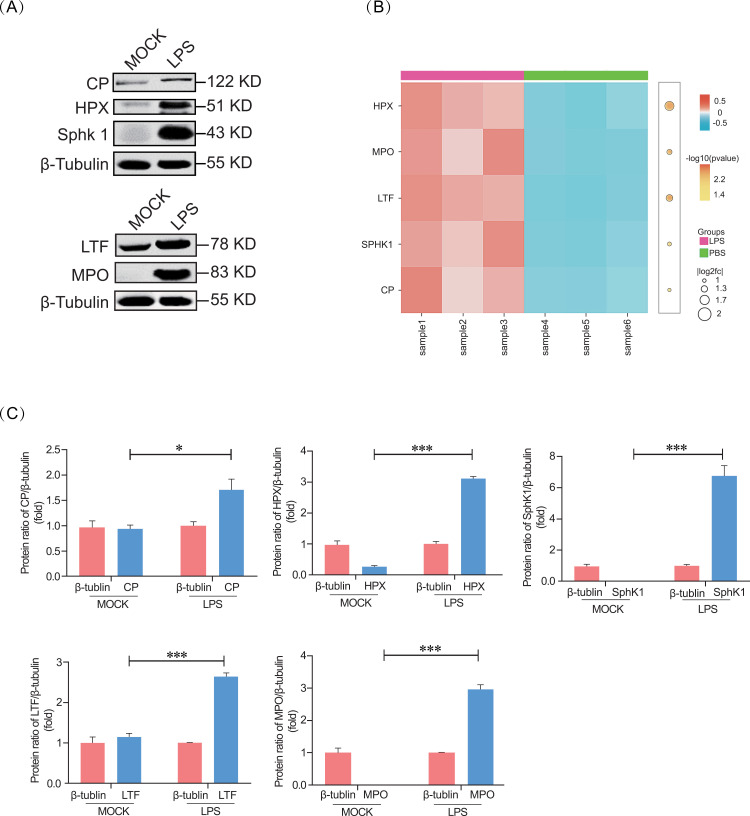
Confirmation of DEPs by western blot. (A) Immunoblotting analysis of proteins (CP, HPX, SphK1, LTF, and MPO) in LPS-induced and PBS tree shrews. The β-tubulin protein was used as a control. (B) Heatmaps (hierarchical clustering) of the 5 proteins between the PBS and LPS groups, respectively. Three biological replicates are shown. (C) Quantitative analysis of A using ImageJ software. * *p* <  0.05, ***p <  0.001 vs PBS.

Given the results of the GO terms, KEGG pathways, and fold change analysis, the five different protein expression levels of ceruloplasmin (CP), hemopexin (HPX), sphingosine kinase 1 (SphK1), lactotransferrin (LTF) and myeloperoxidase (MPO) were selected for further validation. The results showed that the five expression levels of these proteins were remarkably higher in the LPS group than those in the PBS group, which was consistent with the proteomic results. Collectively, the protein expression patterns of all validated proteins obtained from the TMT-based proteomics and western blot analysis identified similar increases in protein expression (Fig 5).

## 4. Discussion

**Fig. 6:**
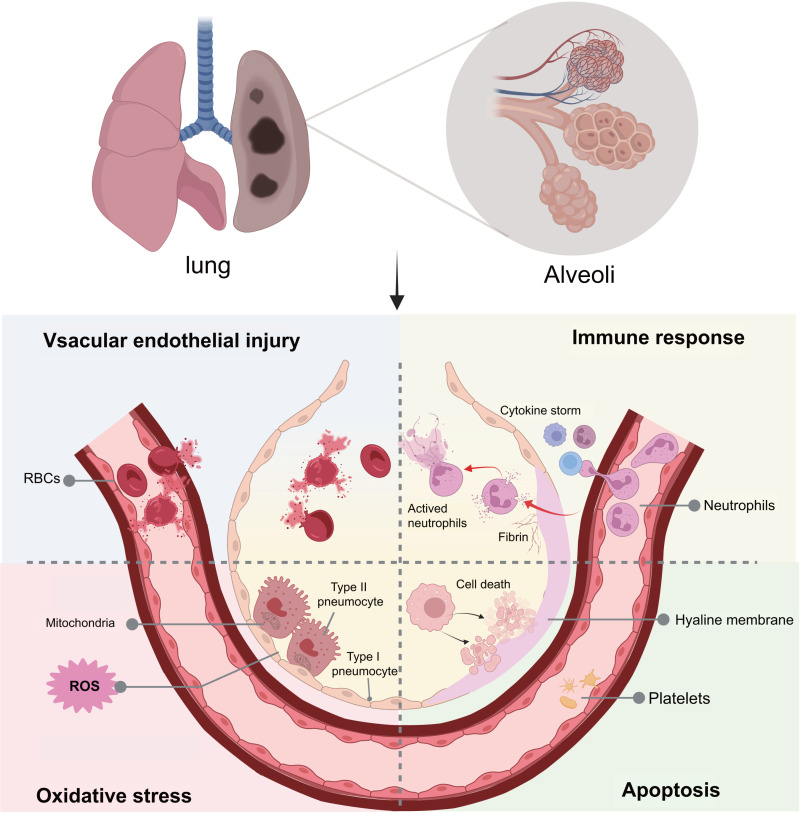
Key pathways and protein profiles in ARDS tree shrews. This figure was created by Biorender (https://app.biorender.com/).

We previously established a ‘one-hit’ LPS-induced ARDS tree shrew model, which proved exceptionally advantageous over other commonly-used rodent species in terms of morphology, clinical symptoms, and irreversible lung injury. However, the molecular changes have not yet been explored in the lungs of tree shrews. In this study, we investigated the pathological lesions and achieved deeper coverage of the lung proteome based on TMT proteomics. By distilling this comprehensive information, we can obtain valuable evidence that clearly reveals the ARDS-related pathways and proteins in the lung tissues of tree shrews and recommend the application of the novel model for preclinical practice (Fig 6).

The results of this study demonstrated a significant increase in total immune cells and injury-related proteins in the lung tissues of tree shrews in response to the LPS challenge. For example, alveolar epithelial type II cells are associated with innate immunity and immune homeostasis in the pathogenesis of various parenchymal lung diseases, which is in accordance with animal models and clinical findings of patients [46–48]. Combined with increased alveolar macrophages, neutrophils, and alveolar epithelial type II cells, which account for the synthesis and release of various inflammatory mediators and influence the development of ARDS following any stimuli [32].

It is worth noting that the significant increase in a series of immune-related genes abundance and the enrichment of proteins associated with LPS induction, which may indicate important roles for these parts of the protein network—such as the dramatically increased proteins HPX, CP, LTF, MPO, and SphK1. Hemopexin (HPX), a plasma protein that binds to heme with special affinity, has been proposed as a possible treatment approach to decrease inflammation in critically ill patients [49]. The above baseline of HPX has been observed in mouse models of burn wound infection, endotoxemia, and peritonitis-induced sepsis [49]. In addition, the increased levels of HPX have been reported as a protective response of ill patients to the acute phase of acute inflammatory disease, which is induced by sepsis [49,50]. In this context, the expression of HPX was upregulated in ARDS tree shrew models confirmed by proteomic method and western blot analysis. However, the role of HPX in the process of ARDS and HPX-driven pathways in this disease needs to be further investigated. ARDS is a severe lung injury caused by extensive inflammation. Lactoferrin (LTF) is a natural immune modulator, which is expressed by cells of the innate immune system and exists in secondary neutrophil granules. Recently, it has become a potential strategy of ARDS, particularly regarding COVID-19 [51–53]. The significant upregulation of LTF in the tree shrew model, as the innate immune system activator, is associated with intravascular granulocyte activation, which may mediate signaling in response to LPS [54]. Meanwhile, myeloperoxidase (MPO), another important activator located in neutrophil granules, is characterized by powerful proinflammatory and pro-oxidative properties [55]. It contributes to the pulmonary endothelial injury process in the sepsis of human plasma or to endotracheal aspirates in sterile inflammation of sepsis-associated ARDS [56,57]. As an index of neutrophil response, it has been widely used in endotracheal aspirates of COVID-19 ARDS patients and clinical patients with ARDS from other causes [58]. Consistent with prior literature, our study presents compelling evidence that MPO is sharply increased in the lung tissues of the ARDS tree shrew group but not in the PBS group. Moreover, it is worth noting that other multifunctional proteins significantly changed in response to LPS in the lungs of tree shrews, which also have been identified involved in ARDS, such as sphingosine kinase 1 (SphK1) and ceruloplasmin (CP). SphK1 has been identified as involved in a wide range of processes, including apoptosis, proliferation, angiogenesis, inflammation, and endothelial barrier function [59]. Prior studies have confirmed that cellular expression of SphK1 proteins is dramatically upregulated in alveolar macrophages, alveolar epithelial cells, and endothelial cells in the lung tissues of ARDS mice compared to those of control groups, which is consistent with our results [60–62]. Ceruloplasmin (CP) is a major circulating antioxidant, and increased levels of bronchoalveolar lavage fluid (BALF) and plasma exudations of ARDS patients were observed [63]. In this study, we found that CP was upregulated in LPS-induced lung tissues of tree shrews, produced in the oxidant-mediated inflammation-damaged tissue, of which the increased levels were consistent with both present in serum and BALF [64]. In addition, the immune response mediator of IL-18 and proinflammatory mediator of IL-1 receptor accessory protein (IL1RAP) were sharply increased in the induced group, which was closely associated with sepsis severity in patients [41–45].

Together, the results in this study support the significant induction of direct or indirect pulmonary inflammatory responses in tree shrews, which may be one of the factors associated with LPS-induced lung injury by engaging the alternatively activated neutrophil pathway.

## 5. Limitations

In the current study, we found that the immune response of the lungs showed several critical ARDS-related proteins and pathways in LPS-induced tree shrews. However, this study has several limitations. Firstly, the exact mechanisms underlying the altered protein profile triggered by LPS instillation will need further investigation; the exact molecular mechanisms of the essential gene expressions that are involved in the immunity of tree shrews still need to be determined. The related pathways they interact—whether they operate upstream, downstream, or parallel to one another—are still unknown. Secondly, we do not understand what the protein expression of other ARDS causes (beyond LPS alone) would be. Further studies need to validate the cellular results in tree shrew models with ARDS established by other causes, which will help reveal the mechanisms linking gene expression and syndromes. Thirdly, tree shrew models remain woefully incomplete models for human disease and unavailable, accompanied by commercial reagents, limiting their application. Therefore, further study combined with other animal models that capture the multifactorial nature of ARDS could provide better insights into the underlying pathophysiology and pathways involved in ARDS.

## 6. Conclusion

In summary, our study investigated pathological lesions as well as protein profiles focused on the immune response of lung tissues and identified the related signal pathways. Some of the reported upregulated proteins that participated in lung function and immune response, involved in other ARDS models, were also observed in our tree shrew ARDS model. These findings enhance our understanding of LPS-induced pathogenesis in tree shrews and can help future studies evaluate the immune response to LPS with other modeling methods in order to achieve a comprehensive and systematic analysis of ARDS tree shrew models. Our work serves as a valuable resource for identifying the protein profiles after intratracheal LPS induction in tree shrews, which may indicate the potential pathophysiology and ARDS-related pathways in tree shrews, thereby promoting a novel animal model for translational research in the ARDS specialty.

## Supporting information

S1 Table Identified and quantified 4,070 proteins. (XLS)

S2 Table Identified and quantified 1.5-fold DEPs. (XLS)

S3 Table GO enrichment analysis of 1.5-fold DEPs. (XLS)

S4 Table Analysis of 1.5-fold DEPs in four key pathways of the human ARDS. (XLS)

## Abbreviations

CP: Ceruloplasmin
HPX: hemopexin
SphK1: sphingosine kinase 1
LTF: lactotransferrin
MPO: myeloperoxidase
ARDS: acute respiratory distress syndrome
ALI: acute lung injury
BALF: bronchoalveolar lavage fluid
HE: hematoxylin and eosin
LPS: lipopolysaccharide
